# Urinary Tract Infection in Pregnancy and Its Effects on Maternal and Perinatal Outcome: A Retrospective Study

**DOI:** 10.7759/cureus.21500

**Published:** 2022-01-22

**Authors:** Lekshmi Balachandran, Leena Jacob, Reem Al Awadhi, Lamia O Yahya, Khlood M Catroon, Lakshmi P Soundararajan, Saleema Wani, Sara Alabadla, Yassmin A Hussein

**Affiliations:** 1 Obstetrics and Gynecology, Corniche Hospital, Abu Dhabi, ARE; 2 Pharmacology and Therapeutics, Corniche Hospital, Abu Dhabi, ARE

**Keywords:** bacteria, recurrent uti, pyelonephritis, lbw, preterm delivery, uti

## Abstract

Background

A urinary tract infection (UTI) is a common medical condition complicating pregnancy with adverse maternal and perinatal outcomes. This study aimed to assess any adverse maternal and perinatal morbidity related to UTI in pregnancy, focusing on identifying common uropathogens and their antibiotic sensitivity and resistance patterns.

Methods

We conducted a retrospective cohort study at Corniche Hospital, Abu Dhabi. The study population consisted of 549 women in the exposed group (i.e., those with at least one episode of UTI in pregnancy in 2018) and 329 in the comparison group (i.e., those without UTI). Statistical analysis was done using SPSS Statistics for Windows, Version 19.0 (SPSS Inc., Chicago, IL). The study's primary outcome measures were preterm birth, recurrent UTI, pyelonephritis, and low birth weight (LBW).

Results

Women who had a UTI during pregnancy had more preterm deliveries than those without a UTI (c2=7.092; p=0.007). Recurrent UTI was observed in 26.6% of women with UTI, while the incidence of pyelonephritis was relatively low in this group (1.45%). There was no significant association between LBW and UTI in pregnancy (c^2^=0.097; p=0.756). The most common bacteria isolated from women with UTI were Group B Streptococcus (GBS, 31.3%), followed by *Escherichia coli* (30.9%). They were sensitive to a wide range of antibiotics.

Conclusion

According to our results, significant predictors of bacteriuria in pregnancy history include UTI, renal calculi, and nulliparity. Women with UTI in pregnancy are more likely to have preterm delivery. However, adequate management can minimize other complications like pyelonephritis and adverse perinatal outcomes. Available evidence prompts the recommendation of routine screening for asymptomatic bacteriuria (ASB) in early pregnancy to minimize complications and identify those women at significant risk for preterm delivery.

## Introduction

Urinary tract infections (UTI) continue to be one of the most common medical conditions complicating pregnancy, with a prevalence of approximately 20% [1]. A UTI is diagnosed when there is an overgrowth of bacteria in the urinary tract (≥105 counts/mL of urine), irrespective of the presence of clinical symptoms [2]. UTI include a spectrum of disorders, ranging from those affecting the lower urinary tract, like asymptomatic bacteriuria (ASB) and cystitis, to those affecting the kidney, such as pyelonephritis. The prevalence of ASB is 2% to 10% of cases [3]. Clinical trials in the 1960s and 1970s reported that untreated ASB had a 20% to 30% risk of progressing into pyelonephritis. Early diagnosis and adequate treatment with antibiotics helped reduce the risk by 80% [4].

Organisms causing UTI in women (whether pregnant or not) are of the same species and virulence factors. Bacteria commonly isolated include *Escherichia coli*, *Klebsiella pneumonia*, Proteus, Acinetobacter, *Staphylococcus saprophyticus*, Group B Streptococcus (GBS), and *Pseudomonas aeruginosa* [5-7].

Advanced maternal age, multiparity, sexual intercourse, diabetes, sickle cell anemia, previous history of UTI, immunodeficiency, and urinary tract abnormalities are risk factors for UTI in pregnancy [6,7]. UTI in pregnancy is considered a risk factor for adverse maternal and perinatal outcomes. Schieve et al. reported an increased association of UTI with premature labor, hypertensive disorders of pregnancy, anemia, and amnionitis [8]. Delzell and Lefevre noted similar outcomes but added low birth weight (LBW) infants as an additional outcome [9]. However, the population-based nationwide study by Chen et al. in Taiwan found no increased risk for LBW, no incidence of neonates small for gestational age, or preterm babies in women with antenatal UTI [10]. Given the conflicting reports in the literature, we conducted this study to look for any adverse maternal and perinatal morbidity related to UTI in pregnancy and identify common uropathogens and their antibiotic sensitivity and resistance patterns.

## Materials and methods

Design

We conducted a retrospective cohort study of pregnant women who received antenatal care between January 1, 2018, and December 31, 2018, at Corniche Hospital, a tertiary-care maternity hospital in Abu Dhabi, United Arab Emirates. The Corniche hospital ethics committee granted ethical approval (Reference No: CH050993) even though the study did not directly involve human subjects.

Sampling

The study group consisted of pregnant women who had at least one positive urine culture during the study period. All nonpregnant patients with UTI (including postpartum UTI), those with miscarriages, and those with preexisting congenital renal tract anomalies and chronic renal diseases were excluded.

The comparison group consisted of pregnant women who attended Corniche hospital for antenatal care during the same period but had no episodes of UTI in pregnancy. Five hundred women booked in the initial few months were selected from the booking data after excluding those with confirmed UTI during pregnancy. The exclusion criteria for the study group were further applied to the final comparison group (n=330).

After applying the exclusion criteria, we collected data from a total of 993 pregnant women, including women with confirmed UTI (n=663) and those without UTI (n=330). In the UTI group, 114 women were excluded from the analysis due to lack of follow-up data or postnatal diagnoses, leaving 549 cases in the exposed group. In the comparison group, one case was excluded for the same reason, leaving 329 women in the control cohort without UTI. A total of 878 women were involved in the final analysis.

Thirty-one women with UTI were diagnosed at advanced gestation of 37 weeks or later in their pregnancy. Therefore, they were excluded from determining the association between UTI and preterm delivery. There were no such exclusions in the comparison group.

We recorded basic demographic data consisting of age, nationality, body mass index (BMI), and parity and the previous history of UTI, comorbidities, gestational age at first diagnosis, and delivery and birth weight of babies. Urine culture results with bacterial isolates, their sensitivity, antibiotics used, and recurrence-related data were obtained from the patients' electronic medical records. In addition to routine screening at booking, cultures were also done when patients presented with symptoms of UTI.

Outcomes

The primary outcome was maternal and perinatal morbidity associated with UTI in pregnancy. Maternal outcomes included those who had premature delivery (i.e., those with the onset of labor and delivery prior to 37 completed weeks of gestation), those with recurrent UTI (i.e., those with more than one episode of UTI in the index pregnancy), and pyelonephritis with clinical features of upper UTI and diagnosed as pyelonephritis in the patients' records. Perinatal morbidity was measured in terms of LBW (<2500 g at ≥37 weeks), prematurity (<37 weeks' gestation), and preterm LBW (birthweight <10th percentile on Fenton's birthweight distribution chart at each gestational age) [11].

The secondary outcome measures included the identification of the common organisms responsible for primary and recurrent infections. We also assessed the type of recurrent infections (whether due to relapse or reinfection) and the antibiotic sensitivity and resistance patterns.

Recurrent UTIs are symptomatic UTIs that occur after the resolution of an earlier episode, usually after appropriate treatment [12]. Recurrent UTIs include relapses (i.e., symptomatic recurrent UTIs with the same organism following adequate therapy) and reinfection (i.e., recurrent UTIs with previously isolated bacteria after treatment and with a negative intervening urine culture, or a recurrent UTI caused by a second bacterial isolate) [13].

Data analysis

We used SPSS Statistics for Windows, Version 19.0 (SPSS Inc., Chicago, IL) to find the association of variables, and statistical tests (i.e., T-test, comparison of means, tests of association, Chi-square, and the test of proportions) were carried out appropriately. Continuous and categorical data were analyzed using descriptive statistics and presented as mean, standard deviation, and percentage. Associations with p<0.05 were considered statistically significant.

## Results

**Figure 1:**
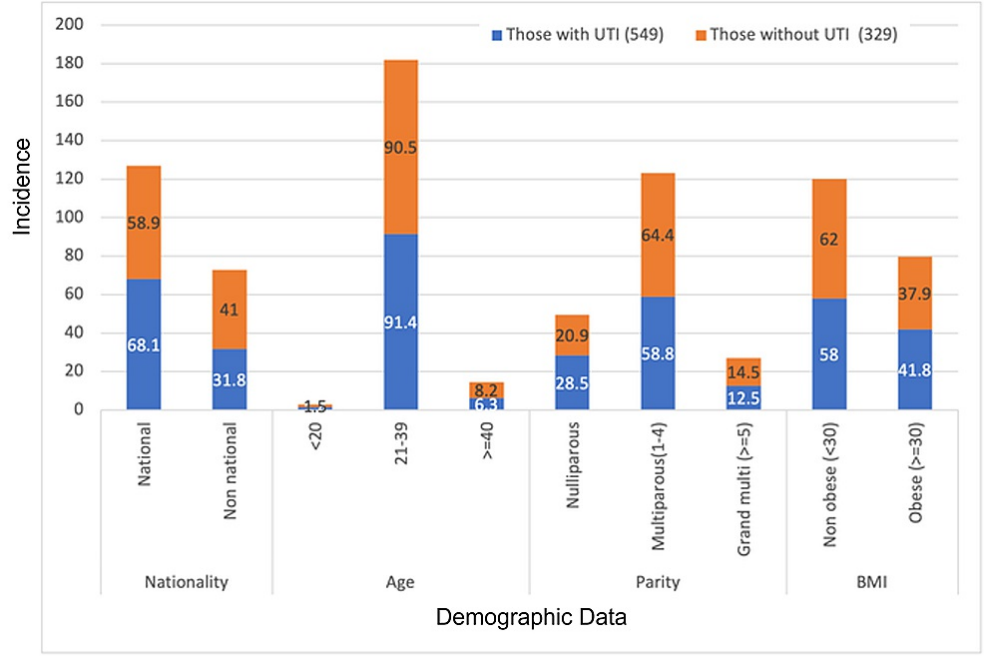
Demographic characteristics BMI: body mass index

In 2018, 4277 pregnant women visited Corniche hospital for antenatal care, with 663 having culture-proven UTI (15% prevalence). Figure 1 depicts the demographic characteristics of the study participants. Most of the women who participated in our study were aged 31 to 40 (49.5%) or 21 to 30 (44%). The mean age of the exposed group was 30.5 years, while that of the comparison group was 31.8 years. There was no significant association noted between age and the risk of UTI (c2=5.971; p=0.113).

The mean BMI of women with UTI was 28.5 kg/m^2^, and the control group's mean BMI was 28.4 kg/m^2^. There was no significant association between BMI and UTI (c2=1.29; p=0.25). However, we noted a significant association between parity and UTI. Urinary infection was more common in nulliparous women than in multiparous women (c2 = 6.337; p =0.042). UTI was more prevalent among UAE nationals as opposed to other nationalities (c2=10.99; p=0.0009). A history of UTI was associated with more cases of UTI in pregnancy than those without a pre-pregnancy UTI (c2=63.881; p=0.0001).

The commonly associated comorbidities like anemia, diabetes, and hypertension did not significantly correlate with UTI (Table 1). However, those with hypothyroidism were more likely to have UTIs in pregnancy (c2=13.68; p=0.0002). All the women with renal calculi (n=18) developed UTI in pregnancy.

**Table 1:** Parity, UTI history, other major comorbidities in patients with UTI *Statistically significant. UTI, urinary tract infection; Hx, history; GDM, gestational diabetes mellitus; PET, preeclampsia; MP, multiple pregnancies; HT, hypertension.

	Control group (n=329)	Exposed group (with UTI; n=549)	Total (n=878)	Chi-square	P-value
Parity					
Nulliparous	69 (21.0%)	157 (28.6%)	226 (25.7%)	6.337	0.042*
Multiparous	212 (64.4%)	323 (58.8%)	535 (60.9%)		
Grand multiparous	48 (14.6%)	69 (12.6%)	117 (13.3%)		
UTI Hx	11 (3.3%)	131 (23.9%)	142 (16.2%)	63.881	0.0001*
GDM	87 (26.4%)	132 (24%)	219 (24.9%)	0.633	0.426
Anaemia	47 (14.3%)	59 (10.7%)	106 (12.1%)	2.427	0.119
PET	7 (2.1%)	11 (2%)	18 (2.1%)	0.016	0.900
Renal calculi	0 (0%)	18 (3.3%)	18 (2.1%)		-
MP	7 (2.1%)	9 (1.6%)	16 (1.8%)	0.274	0.601
HT	2 (0.6%)	5 (0.9%)	7 (0.8%)	0.239	0.625
Hypothyroidism	15(4.5%)	66 (12%)	81 (9.2%)	13.68	0.0002*

One hundred fifteen women (21%) with UTI presented with one or more UTI symptoms. However, most women with UTI (79%) were asymptomatic and identified by routine screening at their visit.

Maternal and perinatal outcomes of UTI in pregnancy

Study participants with UTI had more preterm deliveries compared to those without UTI (c2=7.092; p=0.007; odds ratio [OR], 1.659; 95% confidence interval [CI], 1.142 to 2.408). This was not significantly associated with their parity, age group, BMI, or nationality. Of the associated comorbidities, only hypertension in pregnancy was linked to preterm deliveries. Similarly, gestational age at first diagnosis of UTI was also not associated with gestational age at delivery (c2=0.5432; p=0.76; Table 2). 

**Table 2:** Association of gestational age at diagnosis and preterm delivery

Gestational age at diagnosis	Term delivery	Preterm delivery	Total (n=518)	Chi-Square	P-value
1st trimester	107 (82.3%)	23 (17.7%)	130 (25.1%)	0.5432	0.76
2nd trimester	182 (79.4%)	47 (20.5%)	229 (44.2%)		
3rd trimester	130 (81.7%)	29 (18.2%)	159 (30.7%)		
Total	419 (80.9%)	99 (19.1%)	518 (100%)		

In the exposed group, 146 women (26.6%) had recurrent UTI. Relapse of infection was noted in 71 (48%) of these women, and reinfection in 107 women (73%). Thirty-two women (22%) had both relapse and reinfection. None of the demographic factors or associated comorbidities were associated with recurrent UTI except for those with a previous history of UTI; they were 1.9 times more likely to have a recurrence (c2=8.8974; p=0.002; OR, 1.88; 95% CI, 1.23-2.87). The likelihood of preterm delivery in those with recurrent UTI was not significantly different from that of women with one episode of UTI in pregnancy (c2=0.111; p=0.739). There were eight cases of pyelonephritis (1.45%) in the study group, of which two were preterm deliveries. Because the numbers were low, it was impossible to analyze the data for a significant association.

A small percentage (5.3%) of babies born to mothers with UTI had LBW, while a similar percentage (5.8%) of babies from mothers with no UTI had LBW. Essentially, there was no significant relationship between LBW and the presence or absence of UTI in pregnancy (c2=0.097; p=0.756). Premature babies were more common in women with UTI than in the comparison group (c2=7.092; p=0.007; Table 3).

**Table 3:** UTI association with preterm birth and LBW babies UTI, urinary tract infection; LBW, low birth weight; NA, not applicable.

	Control group (n=329)	Exposed group (after excluding UTI diagnosed at ≥37 weeks; n=518)	Total (n=847)	Chi-square	P-value
Term delivery	289 (87.8%)	419 (80.8%)	708 (83.6%)	7.092	0.007*
Preterm delivery	40 (12.2%)	99 (19.1%)	139 (16.4%)	NA	NA
LBW	19 (5.8%)	29 (5.3%)	48 (5.5%)	0.097	0.756

Pathogens

**Figure 2:**
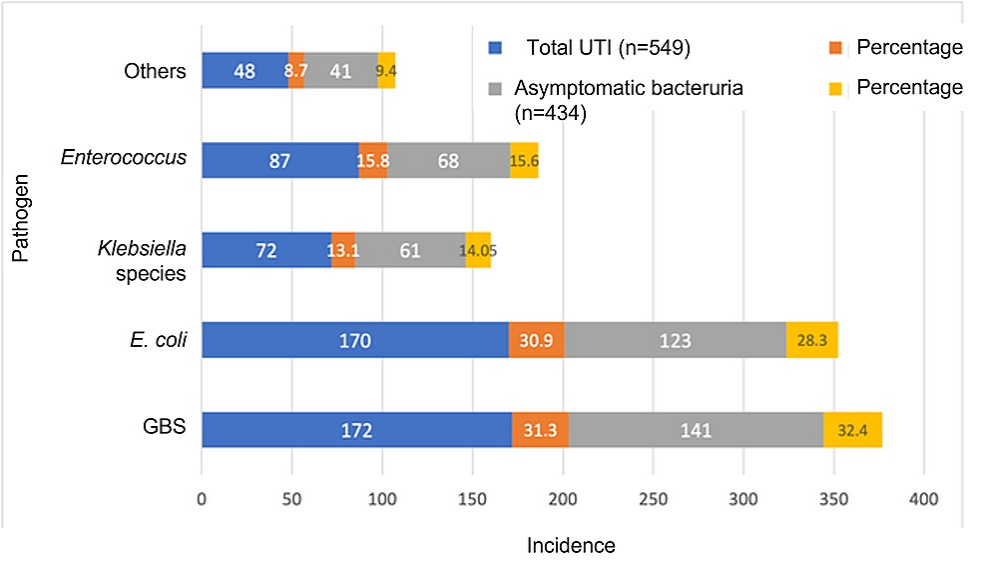
Common uropathogens isolated GBS, Group B Streptococcus; UTI, urinary tract infection.

The most common bacteria isolated from women with UTI were GBS (31.3%), *E. coli* (30.9%), Enterococcus (15.8%), *K. pneumoniae* (13.1%), and other minor groups of organisms (9%; Figure 2). ASB constituted 79% of the confirmed UTI cases. The bacterial isolates in ASB followed the same pattern, with GBS in 32%, *E. coli* in 28%, and Enterococcus and Klebsiella in 15% and 14% of cases, respectively.

The most common organism responsible for recurrent UTI was *E. coli* (40%), with GBS, Enterococcus, and *K. pneumoniae* sharing a majority of the remaining 60%. *E. coli* was also the primary organism causing UTI relapse (n=24; 34%). GBS was 100% sensitive to vancomycin and 99% sensitive to ampicillin, while 42% were resistant to clindamycin. *E. coli* was only 93% sensitive to nitrofurantoin. A portion (24%) of *E. coli* isolates were extended-spectrum beta-lactamase (ESBL)-producing. Of those, 88% were sensitive to nitrofurantoin. The sensitivity of *E. coli* to fosfomycin was 96%, with 92% sensitivity for ESBL-producing isolates. *Enterococcus faecalis* isolates were 100% sensitive to ampicillin, nitrofurantoin, and teicoplanin, with no relevant resistance patterns. *K. pneumonia* isolates were 94% sensitive to ceftazidime, cefotaxime, and co-amoxiclav.

## Discussion

The prevalence of UTI among pregnant women attending Corniche hospital was 15%, with the majority (11.8%) remaining asymptomatic and only 3.2% presenting with one or more symptoms of UTI. This was in contrast to previous studies by Faidah et al. in Saudi Arabia (who reported a 20% prevalence of UTI, of which 12% were symptomatic and 8% were asymptomatic) and Lee et al. (who reported a UTI prevalence of 8.9%, with 4.4% symptomatic UTI and 4.5% with ASB) [14,15]. The increased prevalence of ASB in our population could be attributed to the strict adherence to our hospital policy of getting a urine culture done at booking for every pregnant woman.

The association of demographic factors and UTI has been controversial, with some studies showing increased risk with advanced maternal age and multiparity [16]. However, Hamdan et al. found no significant association [17]. Our study showed nulliparous women to be more likely to develop UTI. Age, BMI, nationality, and gestational age were not associated with UTI development.

In our study, a history of UTI was significantly associated with an increased risk of UTI. Similar findings were noted by Pastore et al., who identified the two strongest predictors of bacteriuria in prenatal care as antepartum UTI prior to prenatal care and a pre-pregnancy history of UTI [18].

Hypothyroidism had a significant association with UTI in pregnancy in our study but has not been reported as such, according to a literature search. Only one case was reported by Correia et al. of an association between subclinical hypothyroidism and recurrent UTI in a nonpregnant woman [19]. In the absence of additional evidence, further studies are needed to ascertain the significance.

Another significant outcome of this study was the association between preterm delivery and UTI. Similar outcomes were reported by Shieve et al. and Siakwa et al. [8,20]. In 1989, a meta-analysis published by Romero et al. concluded that non-bacteriuric patients had only approximately two-thirds the risk of LBW and half the risk of preterm delivery compared to those with untreated symptomatic bacteriuria, and antibiotic treatment reduced the risk of LBW [21]. Our study differed from this meta-analysis because, despite the fact that 89% of women with UTI received appropriate antibiotic treatment, the risk of preterm delivery remained high. However, this did not translate into a significant increase in LBW babies. Findings similar to our study were reported by Smaill et al., who noted that antibiotic treatment for ASB reduced the risk of pyelonephritis (relative risk [RR], 0.23; 95% CI, 0.13 to 0.41) and LBW (RR, 0.66; 95% CI, 0.49 to 0.89), although no significant reduction in the rates of preterm birth was demonstrated [22]. 

Several studies have shown *E. coli* and other Gram-negative isolates (namely the Klebsiella species, *Acinetobacter baumannii*, and *Proteus mirabilis*) to be responsible for 70% to 80% of UTI in pregnancy. Gram-positive organisms (e.g., *Enterococcus faecalis* and GBS) were isolated in approximately 10% of UTIs in pregnant women [23,24]. However, our study revealed a very high prevalence of GBS bacteriuria (about 30%) in symptomatic and asymptomatic UTIs, followed closely by *E. coli*. Geographic variation with resultant differences in cultural and behavioral practices could account for the deviation. The identification of *E. coli* as the most common bacteria responsible for recurrent UTI, particularly relapse, could be explained by the increased susceptibility to vaginal colonization by the adjacent rectal flora and the inherent affinity for uropathogenic coliforms to adhere to uroepithelial cells [12].

Of the different antibiotics studied, ampicillin was very effective against GBS and Enterococcus species. Fosfomycin has evolved as an effective first-line treatment for *E. coli* strains, including ESBL-positive ones. Similar findings were reported by Rosana et al. [25]. Nitrofurantoin is comparatively less effective against *E. coli*; however, it is 100% effective against Enterococcus species. The very high resistance to clindamycin in GBS UTI limits its viability as an alternative in penicillin-allergic patients. As recommended by the 2010 Centers for Disease Control and Prevention guidelines [26], vancomycin with 100% sensitivity becomes the preferred alternative in this situation.

Limitations

Our study was limited by its retrospective nature, resulting in information bias due to missing data and selection bias due to loss of follow-up. Despite this limitation, our study featured patients of similar demographics in both the UTI and comparison groups.

## Conclusions

Based on our study results, a history of UTI, renal calculi, and nulliparity are significant predictors of bacteriuria in pregnancy, a large portion of which is asymptomatic. Women with UTI in pregnancy are more likely to have preterm delivery. However, adequate management can minimize other complications like pyelonephritis and adverse perinatal outcomes. Our study emphasizes the importance of routine screening for ASB in pregnancy rather than screening only those women with symptoms. This approach would help identify high-risk women at significant risk for preterm delivery, allowing targeted care and proper use of available resources. On the other hand, widespread injudicious use of antibiotics and the subsequent development of antibiotic resistance are growing concerns. Therefore, health care teams should consider regular reviews of antibiograms and choose the appropriate antibiotic in each case.
